# Photostable and Uniform CH_3_NH_3_PbI_3_ Perovskite Film Prepared via Stoichiometric Modification and Solvent Engineering

**DOI:** 10.3390/nano11020405

**Published:** 2021-02-05

**Authors:** Daocheng Hong, Mingyi Xie, Yuxi Tian

**Affiliations:** 1 Key Laboratory for Advanced Technology in Environmental Protection of Jiangsu Province, Yancheng Institute of Technology, Yancheng, Jiangsu 224051, China; hdaocheng@gmail.com; 2 Key Laboratory of Mesoscopic Chemistry of MOE, School of Chemistry and Chemical Engineering, Nanjing University, Nanjing, Jiangsu 210023, China; xmy@nju.edu.cn

**Keywords:** CH_3_NH_3_PbI_3_, stoichiometric modification, morphology, photostability, solvent engineering

## Abstract

Solution-processed organometal halide perovskites (OMHPs) have been widely used in optoelectronic devices, and have exhibited brilliant performance. One of their generally recognized advantages is their easy fabrication procedure. However, such a procedure also brings uncertainty about the opto-electric properties of the final samples and devices, including morphology, stability, coverage ratio, and defect concentration. Normally, one needs to find a balanced condition, because there is a competitive relation between these parameters. In this work, we fabricated CH_3_NH_3_PbI_3_ films by carefully changing the ratio of the PbI_2_ to CH_3_NH_3_I, and found that the stoichiometric and solvent engineering not only determined the photoluminescence efficiency and defects in the materials, but also affected the photostability, morphology, and coverage ratio. Combining solvent engineering and the substitution of PbI_2_ by Pb(Ac)_2_, we obtained an optimized fabrication condition, providing uniform CH_3_NH_3_PbI_3_ films with both high photoluminescence efficiency and high photostability under either I-rich or Pb-rich conditions. These results provide an optimized fabrication procedure for CH_3_NH_3_PbI_3_ and other OMHP films, which is crucial for the performance of perovskite-based solar cells and light emitting devices.

## 1. Introduction

Organometal halide perovskites have attracted a great deal of attention and become one of the most promising photovoltaic and optoelectronic materials [1,2,3] due to their brilliant optoelectronic properties, including a large absorption coefficient [4,5], long charge carrier diffusion length [6,7,8,9,10], and low exciton binding energy [11,12,13]. Rapid progress, such as photovoltaic devices with power conversion efficiency over 25.5% [14] and light-emitting diodes with external quantum efficiency exceeding 20% [15,16], have been achieved in just a few years. However, there are still many debates that exist about these fast proceeding processes. As is well known, the electronic structure of CH_3_NH_3_PbI_3_ is insensitive to a large range of compositional changes [17,18], due to its high defect tolerance properties, which determined the high stoichiometric tolerance during the fabrication process. However, arguments about whether an excess of PbI_2_ or CH_3_NH_3_I will be beneficial for improving the performance of the perovskite materials [19,20,21,22] never went away. Different kinds of mechanism, responsible for the improvements, have also been proposed by investigators.

Son et al. reported that adding excess CH_3_NH_3_I to the precursor solution and depositing by means of a Lewis acid-base adduct approach can effectively suppress non-radiative recombination at the grain boundaries [23]. Yang et al observed that the introduction of additional iodine ions into the organic cation solution can decrease the concentration of deep-level defects [24]. Conversely, Chen et al. suggested that a proper amount of PbI_2_ species in the grain boundaries can lead to an improved carrier behavior [25]. That remnant lead iodide can reduce the halide vacancy concentration and enhances the performance of perovskite solar cells was proved by Park et al. [26]. Detailed comparisons have also been made by Jacobsson et al.; they proposed that PbI_2_-rich perovskite film solar cells showed better performance for effective photocurrent enhancement, while PbI_2_-deficient samples can prepare high-quality crystals [22].

Thus, we intended to study the stoichiometric influences on CH_3_NH_3_PbI_3_ films to reveal the detailed differences. In this work, by using wide-field fluorescence microscopic technology, we observed that a high coverage of the CH_3_NH_3_PbI_3_ film will be readily available on the substrate via anti-solvent washing-steps during the spinning process [27,28,29], with the increase of PbI_2_ proportion. However, by comparing the photoluminescence (PL) efficiencies of the CH_3_NH_3_PbI_3_ films, a small amount of excess CH_3_NH_3_I in CH_3_NH_3_PbI_3_ films will be beneficial for improved PL efficiencies. Moreover, the photostability of the PbI_2_-rich CH_3_NH_3_PbI_3_ crystals will also be decreased, which will greatly limit their service life when applied in optoelectronic devices. To obtain an uniform film, with relatively high coverage, PL efficiencies, and photostability, about a 15% excess of CH_3_NH_3_I was required in precursor solutions. In addition, if it is necessary to obtain Pb-rich CH_3_NH_3_PbI_3_ films with high photostability, Pb(Ac)_2_ can be introduced to replace PbI_2_ as the excess Pb source, to fabricate Pb-rich CH_3_NH_3_PbI_3_ films, and which can effectively improve the photostability of the films. In general, the stoichiometric adjustment was necessary for the optimization of the OMHPs films, and had a non-negligible effect on improving the working durability of the OMHPs-based optoelectronic devices.

## 2. Materials and Methods

### 2.1. Material Preparation

Lead iodide (PbI_2_) and lead acetate were separately purchased from Alfa Aesar (Ward Hill, MA, USA) and p-OLED (Xi’an, Shanxi, China). Methylammonium iodide (CH_3_NH_3_I) was purchased from Heptachroma (Yingkou, Liaoning, China). N, N-Dimethylformamide (DMF), dimethyl sulfoxide (DMSO), and chlorobenzene were all purchased from Aladdin (Shanghai, China). For precursors dissolved in DMF, the equimolar stoichiometry (molar ratio of CH_3_NH_3_I and PbI_2_ was 1:1) precursors were first prepared as reported [30]. The over-stoichiometry and under-stoichiometry solutions were produced separately by adding CH_3_NH_3_I and PbI_2_ solutions, and finally diluted to 0.33 mol/L. For mixed solvents, DMF and DMSO were mixed by a volume ratio of 6:1. Then the preparation steps were the same as mentioned above. The Pb(Ac)_2_ was also introduced by adding the solutions into the equimolar stoichiometric CH_3_NH_3_PbI_3_ solutions, and then being diluted to 0.33 mol/L. The solution was coated onto the cover glass by a consecutive two-step spin-coating process at 1000 and 3000, for 5 and 30 s, respectively. Films dealing with solvent engineering were made by dropping 100 μL chlorobenzene onto the substrate during the second spin-coating step. After the spin-coating, the film was annealed at 80 °C for 20 min to achieve formation of perovskite crystals on the coverslips.

### 2.2. Characterizations

XRD measurements were carried out on a X-ray powder diffractometer (Brucker, D8 ADVANCE, Bruker Corporation (China), Guangzhou, Guangdong, China) with Cu Kα radiation (λ = 1.5418 Å) between 10°and 50°, at a scanning rate of 5°·min^−1^.

### 2.3. PL Measurement

The PL of the CH_3_NH_3_PbI_3_ films was analyzed by a home-built wide-field microscope. The excitation source was a 532 nm diode CW laser, focused above the sample plane by a dry objective lens (Olympus LUCPlanFI 40×, NA = 0.6 (Olympus (China) Co., LTD., Beijing, China)). The luminescence signal was collected by the same objective lens, and detected by an EMCCD camera (Andor, iXon Ultra 888 (Andor Technology (UK), Belfase, UK)) after passing through a 550 nm long-pass filter (ET500lp, Chorma (Chroma Technology Crop., Bellows Falls, VT, USA.)). The PL traces were measured by taking movies consisting of 400 frames, and the exposure time per image was 100 ms. PL spectra measurement was made by putting a transmission grating (Newport, 150 lines/mm (MKS Instruments, Inc., Andover, MA, USA)) in front of the camera. The atmosphere was controlled by putting the sample in a chamber filled with nitrogen gas flow.

## 3. Results and Discussions

**Figure 1:**
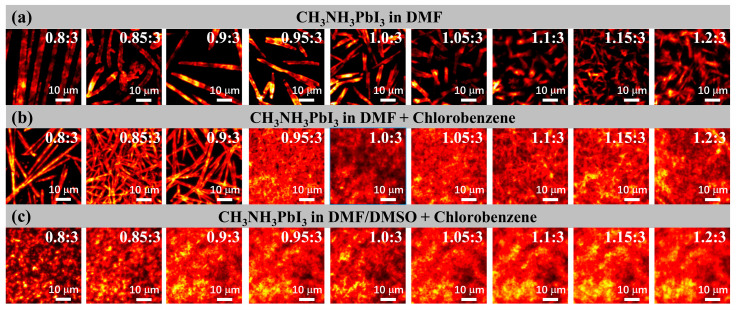
The PL morphology of films composed of different precursors, fabricated via different methods. (**a**) The image of CH_3_NH_3_PbI_3_ films deposited by direct spin without any solvent dropping, and the precursors dissolved in DMF, (**b**) and (**c**) were spun with chlorobenzene dropping, and the precursors were separately dissolved in DMF and DMF/DMSO mixed solvent.

As is well known, dense and uniform OMHP films are the basic requirements for high performance optoelectronic devices, and several methods, including solvent engineering [29,31] and Lewis base adduct theory [32,33], have been suggested for optimizing the absorber layers. Due to the solubility of both precursors being different, and the coordinative effects between PbI_2_ and the solvents also being non-negligible, we first investigated whether different stoichiometric precursors had any influence on the morphology of films deposited via different methods. Here, three different kinds of method were used to prepare the CH_3_NH_3_PbI_3_ films, and the morphology was compared directly via PL images. Figure 1a shows the films in which the precursors were dissolved in N, N-Dimethylformamide (DMF) and deposited via direct spin-casting. Among the films composed of a different precursor ratio, even though the grains became smaller and more dispersed with the increase of the PbI_2_ proportion, the morphologies still all showed low coverage and ribbon crystals, which were ascribed to the different solubilities of PbI_2_ and CH_3_NH_3_I in DMF [32]. To improve the coverage of the CH_3_NH_3_PbI_3_ films, a solvent engineering method was used. After the spread of CH_3_NH_3_PbI_3_ solutions, nonpolar chlorobenzene was dropped onto the substrate during the spinning processes to accelerate the precipitation of the crystals. We observed that the film become dense and homogeneous with the increase of PbI_2_ proportion in precursor solutions, see Figure 1b. However, with the increase of the CH_3_NH_3_I proportion, rod-shaped crystals formed, and the ratio of the coverage decreased. Such results could be due to excessive CH_3_NH_3_I being precipitated together with CH_3_NH_3_PbI_3_ crystals, and evaporated under annealing processes, which finally resulted in the poor coverage. As a result, excessive CH_3_NH_3_I (larger than 15%) is unfavorable for fabricating smooth and dense films.

Furthermore, according to the complex effect between PbI_2_ and Lewis base dimethyl sulfoxide (DMSO), solvent engineering combined with Lewis base adduct was developed. As introduced by Ahn et al., CH_3_NH_3_I, PbI_2_ and DMSO can form CH_3_NH_3_I·PbI_2_·DMSO adducts, and greatly enhanced the solubility of PbI_2_ [32]. According to this theory, we dissolved precursors into DMF/DMSO (volume ratio 6:1) mixed solvents and deposited CH_3_NH_3_PbI_3_ films by washing with chlorobenzene while spinning. The morphology became homogeneous and smooth even in the precursors containing excessive CH_3_NH_3_I, and the grain size also became smaller compared with the films fabricated via the other two methods, as shown in Figure 1c, and could have benefited from the formation of CH_3_NH_3_I·PbI_2_·DMSO intermediates in the precursor solutions. To make this clear, the coverage of the three methods was compared and put together in Table S1. As a result, whichever fabrication method was used, the stoichiometric modifications could all effectively influence the coverage, morphology, and grain size of the perovskite-based films. In addition, the dropping of nonpolar solutions during the spinning process helped to precipitate the intermediates, and promote the formation of homogeneous CH_3_NH_3_PbI_3_ films during the annealing process. The excessive CH_3_NH_3_I could deposit on the surface of the CH_3_NH_3_PbI_3_ films, and did not affect the integrity of the films. Moreover, the thickness of the films was affected by many different experimental conditions including the concentration of the precursor, solvents, temperature, and spin-casting speed, thus the thickness of the films varied from sample to sample. According to the conditions we used in this work, the thickness of the films fabricated via different methods were estimated to range from 500 nm to 2 μm, which should be suitable for optoelectronic devices.

**Figure 2:**
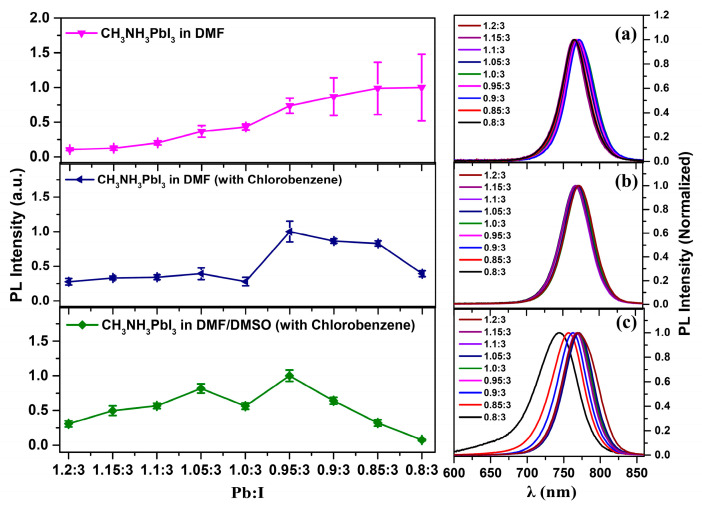
The PL Intensity and spectra of CH_3_NH_3_PbI_3_ films composed of different precursor ratios, via different fabrication methods. (**a**) Precursors dissolved in DMF and spun without any solvent dropping. The excitation was at 450 nm and with a power of 5 W/cm^2^. (**b**) Precursors dissolved in DMF and spun with chlorobenzene dropping. The excitation was at 532 nm, with a power of 4.7 W/cm^2^. (**c**) Precursors dissolved in DMF/DMSO and spun with chlorobenzene dropping. The excitation was at 532 nm, and with a power of 5.5 W/cm^2^.

Besides the influences on film morphology, many groups have also reported that excess PbI_2_ or CH_3_NH_3_PbI_3_ may introduce defect levels or passivate the traps presented in the grain boundaries, which can reduce or improve the performance of OMHPs [34,35,36,37,38,39]. To investigate if stoichiometric adjustment was correlated with the introduction or passivation of the traps in films, the PL properties of the films prepared via different methods, and with a series of precursor ratios, were further compared. Figure 2a shows the PL intensity and spectra of the film fabricated without any solvent dealing. The PL intensity increased with the introduction of the CH_3_NH_3_I in proportion to the precursor solutions indicating a reduction of defects in the CH_3_NH_3_PbI_3_ films, which also coincides with the results reported previously [40,41,42]. Furthermore, the PL spectra showed no obvious shift compared with the band gap transition of CH_3_NH_3_PbI_3_ perovskite [43,44]. The slight fluctuations around 760 nm were likely due to the size-effect of the crystal [45,46]. After changing the preparation method to anti-solvent dropping, the PL spectra could still keep the same as the variation of the precursor. However, the trend of the PL intensity changed in the CH_3_NH_3_I-rich regions, and showed a rapid decrease when the amount of CH_3_NH_3_I excess was over 50%, as shown in Figure 2b. As the PL intensity of PbI_2_-deficient crystals was on the whole higher than that of the PbI_2_-rich crystals, the passivation effect of the CH_3_NH_3_I could still be confirmed. The discrepancy in PL intensity trends suggests a different formation mechanism for nonpolar solvent accelerated the precipitation method compared with the natural growth methods. That is to say, during the natural precipitation process, excess CH_3_NH_3_I can help to improve the quality of the crystals, while in the fast precipitation process, too much CH_3_NH_3_I (>50%) cannot promptly participate in the formation of CH_3_NH_3_PbI_3_ crystals, and may even result in more defects, or other effects on the crystal surface, reducing the PL efficiency of CH_3_NH_3_PbI_3_ crystals. Such results coincided with those reported by Zhang et al. [19], through a small increase in CH_3_NH_3_I, molar ratio (5%), the power conversion efficiency of solar cell devices can improve from 14.06% to 18.26%.

Correlations between the precursor ratio and PL properties of the films fabricated via solvent engineering combined with Lewis base adduct were also characterized. Figure 2c shows that in PbI_2_-rich samples, the PL efficiencies increased with a decrease of PbI_2_, while those in CH_3_NH_3_I-rich samples showed the opposite trend. We suggested that this could be due to the complex effect among CH_3_NH_3_I, PbI_2_, and DMSO, which may require a suitable precursor ratio range to promote the formation of the optimized crystals. In addition, when the amount of CH_3_NH_3_I exceeded 33%, the blue shift of the PL spectra was also observed in CH_3_NH_3_PbI_3_ crystals, indicating a variation of the crystal structure [47,48] under such preparation conditions. That is to say, compared with the solvent engineering method, the adduct of Lewis base will make the sample become more sensitive to excess CH_3_NH_3_I (more than 33%). However, it is worth noting that via this method, either with a small amount of excess PbI_2_ (less than 5%) or CH_3_NH_3_I (less than 15%), the precipitated samples will exhibit a higher PL efficiency than the equal stoichiometric sample. Such results may help to explain why there are some reports that PbI_2_-richness can improve the efficiency [22,25,26], while some claim that PbI_2_-deficiency will enhance the device properties [19,20,49]. Actually, these two states both can improve the performance of the crystals. What needs to be reinforced is that the PL intensity was measured after 20 s light-soaking processing, as there may be PL enhancement or decline behaviors in perovskite materials when under photo-excitation [50,51,52].

**Figure 3:**
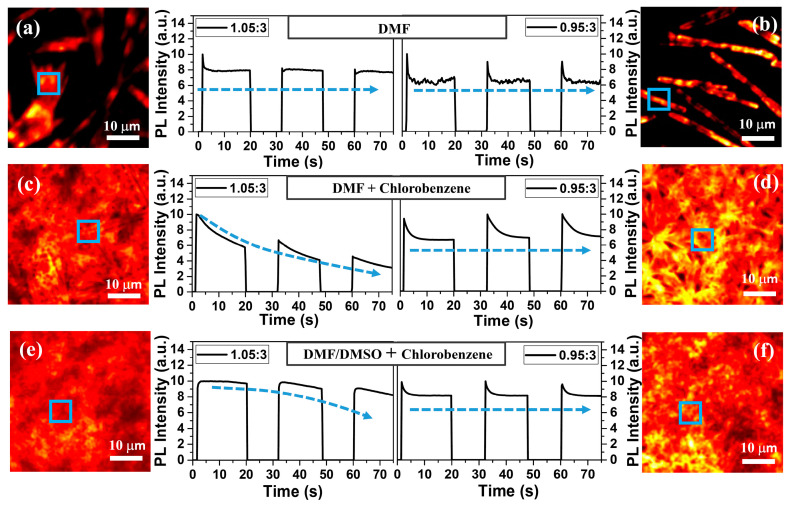
PL morphology and traces of different samples. During the interval in each PL trace, the sample was kept in the dark. (**a**,**b**) were prepared by spinning the precursors dissolved in DMF, (**c**,**d**) were prepared by spinning with chlorobenzene dropping, and the precursors were also dissolved in DMF, (**e**,**f**) were prepared by spinning with chlorobenzene dropping, and the precursors were dissolved in DMF/DMSO. (**a**,**c**,**e**) were prepared from PbI_2_-rich (5%) solutions, and (**b**,**d**,**f**) were prepared from PbI_2_-deficient (5%) solutions. The blue squares in the PL images indicated the measured regions. The excitation power density was 5 W/cm^2^, the excitation wavelength was 532 nm.

Besides the difference in PL efficiencies, there have also been reports about the photo-decomposition mechanism of over stoichiometric (Pbi_2_-rich) samples [22]. Thus, we also tried to check the PL traces of the different stoichiometric films prepared via different methods, to investigate if there were any influences on the photostability properties. Since the PL intensity of CH_3_NH_3_PbI_3_ crystals was vulnerable to the ambient atmosphere, e.g., moisture and oxygen [51,53,54,55], a nitrogen flow was used to avoid their influence during the measurements. We first monitored the PL traces of the samples prepared via direct spin-coating methods. Here, we took a PbI_2_-rich sample with an excess amount of 5%, and a CH_3_NH_3_I-rich sample with an excess amount of 15% as representatives for comparison. As shown in Figure 3a,b, the PL variations of both PbI_2_-rich and CH_3_NH_3_I-rich samples can come to a stable PL intensity in several seconds. Notably, the fast reversible PL decline process during the initial several seconds was due to the reversible conversion of the Pb interstitial defects in the CH_3_NH_3_PbI_3_ crystals between deep-lying and shallow-lying states, as previously reported [52]. Different fabrication conditions, including different preparation methods and different stoichiometric compositions, will influence the concentration of Pb interstitial defects and other defects in CH_3_NH_3_PbI_3_ crystals. The synergistic effects of these defects under photoexcitation will lead to different PL kinetics in different films. Other samples with more or less of an excess of PbI_2_ or CH_3_NH_3_I can also quickly reach a stable PL emission state, as shown in Figure S1. Such results indicated that different stoichiometric CH_3_NH_3_PbI_3_ crystals prepared via direct spin-coating methods can all keep a relatively high photostability, which could be because naturally precipitated crystals are inclined to have better crystallinity. However, when changing the preparation method to nonpolar solvent processing methods, with or without the formation of Lewis base adduct, PbI_2_-rich samples with an excess amount of 5% all showed PL attenuation, as shown in Figure 3c,e, while the PL emission of the samples with an excess amount of 15% CH_3_NH_3_I quickly came to a stable state under continuous photo-excitation. Moreover, PL variations of the samples with other precursor ratios, prepared via these two methods, are also shown in Figures S2 and Figure S3. Summarized results are also presented in Table S2. All the results support that excessive PbI_2_ can reduce the photostability of CH_3_NH_3_PbI_3_ crystals. The attenuation of the sample prepared by solvent engineering method without Lewis base adduct was much faster. We suggested that solvent engineering will promote the precipitation of crystals, which can introduce unreacted amorphous PbI_2_ into perovskite grains or inside the crystals, leading to a reduction of the crystallinity [22]. When without the Lewis base adduct effect, the relative low solubility of PbI_2_ in DMF solvent will make this effect more significant, thus accelerating the attenuation. In general, the direct spinning method can help to prepare CH_3_NH_3_PbI_3_ crystals with improved photostability, whatever the excess amount of PbI_2_ or CH_3_NH_3_I, but fail to obtain films with high coverage. The solvent engineering method with excess PbI_2_ can obtain dense films, but fails to sustain brilliant photostability. Moreover, solvent engineering with Lewis base adduct also showed similar issues in PbI_2_-rich films. To prepare dense films with improved photostability and PL efficiencies, a small excess of CH_3_NH_3_I (less than 15%), and a solvent engineering method with Lewis base adduct were required.

**Figure 4:**
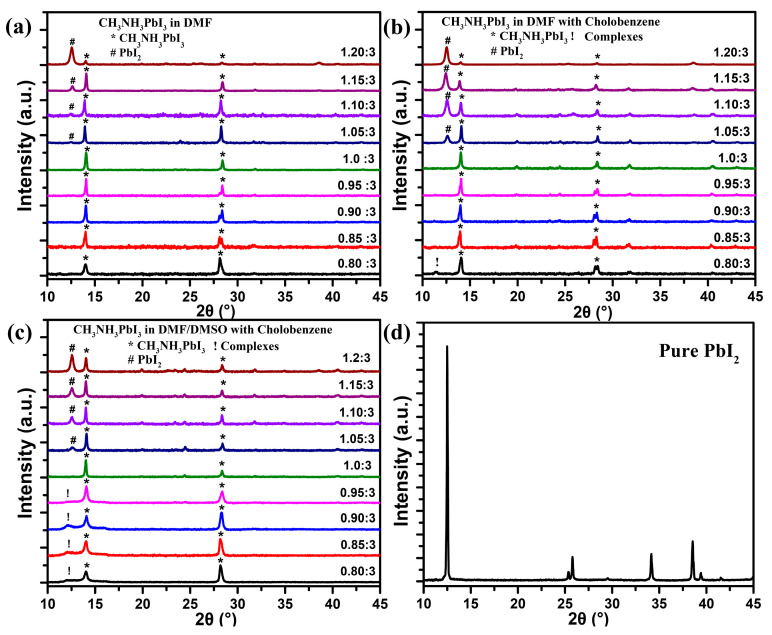
XRD patterns of CH_3_NH_3_PbI_3_ films composed of different precursors ratios. (**a**) Precursors dissolved in DMF, and spun without any solvent dropping. (**b**) Precursors dissolved in DMF and spun with chlorobenzene dropping. (**c**) Precursors dissolved in DMF/DMSO and spun with chlorobenzene dropping. (**d**) Pure PbI_2_ powders were purchased from Alfa Aesar.

To further reveal what effects an excess of PbI_2_ or CH_3_NH_3_I can bring to the crystals, respectively, via these three preparation methods and their correlations with the PL properties, X-ray diffraction (XRD) measurements were taken to investigate the formation mechanisms. Figure 4a shows the CH_3_NH_3_PbI_3_ films prepared via direct spinning methods; we can observe that with an 20% excess of PbI_2_, the signal corresponding to CH_3_NH_3_PbI_3_ peaks was very weak, indicating the poor crystallinity. Additionally, the intensive signal located at 12.6° also proved that the content of the formed CH_3_NH_3_PbI_3_ was less than that of PbI_2_ in the fabricated films [56]. Then, with a decrease of the excessive doped PbI_2_, the signal of CH_3_NH_3_PbI_3_ gradually increased, which can also illustrate the improvement of crystallinity. Furthermore, as reported by Walsh et al. [57], the charged vacancy formation energy of CH_3_NH_3_PbI_3_ perovskite was relatively small. Thus, the excess PbI_2_ could lead to more vacancy defects, such as iodine vacancies, which will cause a decrease of the PL efficiencies. This can explain why more CH_3_NH_3_I can induce the increase of the PL efficiency, as shown in Figure 2a. After introducing a nonpolar solvent, the peak of the PbI_2_ signal became much more intensive, when the excess amount of PbI_2_ was only 5%, as shown in Figure 4b. This result also corresponded to what has been discussed before. During solvent engineering processes, a low solubility will advance the precipitation of PbI_2_ in perovskite grains or inside the crystals, and excess PbI_2_ will induce more deep-lying defects in crystals and produce more photoinduced heat in the crystals [58], leading to the poor photostability, which can also explain the rapid decrease of the photostability, with only a 5% excess amount of PbI_2_. In addition, when there was a 75% excess amount of CH_3_NH_3_I in the precursors (75% excess amount corresponding to Pb/I ratio of 0.8/3, which equaled a PbI_2_/CH_3_NH_3_I ratio of 1:1.75), a small peak at 11.45° appeared, which could be attributed to the presence of solid-state iodoplumbate complexes in the films [47,48]. Formation of such complexes could also be the reason for the rapid decrease of PL efficiencies, existing when the amount of CH_3_NH_3_I excess was over 50% (Figure 2b). Finally, films prepared via solvent engineering processes with Lewis base adducts were also compared. The peak of PbI_2_ also appeared (Figure 2c), but the proportion of the signal was less than that without Lewis base adducts under same stoichiometric circumstances (PbI_2_/CH_3_NH_3_I). This could be due to the strong ability of the Lewis base adduct to coordinate with PbI_2_ to gain the flexibility of tuning coordination strength, which is beneficial for the perovskite formation process [59]. Furthermore, it can also help to passivate the deep-lying defects, improving the photostability. Thus, the attenuation of the PL intensity still cannot be avoided, but the speed was slowed down. However, it seems that the iodoplumbate complexes were much more likely to form with the increase of CH_3_NH_3_I in precursor solutions, as shown in Figure 4c. When the amount of CH_3_NH_3_I excess was over 33%, the broad peak located at 11.45° became more and more obvious. Coincidentally, the PL spectra also started to blue shift from this PbI_2_/CH_3_NH_3_I ratio, as mentioned before (Figure 2c). As a result, we can explain that a certain amount of iodoplumbate complexes forming in CH_3_NH_3_PbI_3_ crystals can be responsible for the variation of the crystal structure, and inducing the PL shift. As a whole, from the analysis above, we can conclude that when the precursors were only composed of CH_3_NH_3_I and PbI_2_, an ideal CH_3_NH_3_PbI_3_ film should introduce a small excess of CH_3_NH_3_I (less than 15%), and be prepared via a solvent engineering method with Lewis base adduct. That is, stoichiometric optimization of the perovskite films can be a key factor for improving the performance and durability of optoelectronic devices, e.g., light-emitting devices and photovoltaic devices.

The above conclusions mainly focus on fabricating iodine-rich samples, while sometimes Pb-rich samples are also necessary. The optimization of performance is also involved with the properties between the perovskite and transport layers [60]. A hypotheses of excess PbI_2_ being beneficial for surface passivation through decreasing hole recombination has been proposed [61]. While, an enhanced electron injection function of PbI_2_ has also been suggested [50]. There are also views of PbI_2_ working as blocking layers which facilitate hole injections and decrease the radiative recombination [25,62]. Theoretical computations have even indicated that iodine-poor conditions can lead to less bulk defects related to deep traps [39]. Overall, lead-rich conditions may also facilitate the improvement of the photovoltaic devices by other paths. However, according to the PL traces characterized above, the Pb-rich films were more vulnerable to photo-irradiation. This poor photostability will limit commercial application.

**Figure 5:**
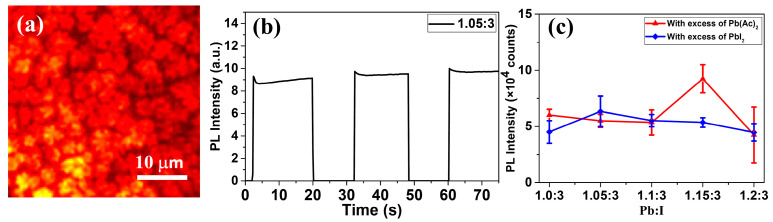
(**a**) PL images of Pb(Ac)_2_-excess CH_3_NH_3_PbI_3_ films, (**b**) PL traces under 532 nm photo-excitation with a power density of 5 W/cm^2^, (**c**) Comparisons of the PL intensity between PbI_2_-excess and Pb(Ac)_2_-excess films.

To solve this issue, we tried to replace Pb(Ac)_2_ with PbI_2_ to create a Pb-rich environment. According to the literature [63], the addition of Pb(Ac)_2_ can accelerate the crystal growth and help to form pin-hole free perovskite films with high crystallinity. In this work, we found that substitution of PbI_2_ by Pb(Ac)_2_ can also improve the photostability of the Pb-rich CH_3_NH_3_PbI_3_. Figure 5a shows the PL morphology of Pb(Ac)_2_-excess CH_3_NH_3_PbI_3_ films, and it seemed that excess Pb(Ac)_2_ could form high-quality crystals, which was consistent with the spiral growth mechanism [64]. Under photo-irradiation, no PL attenuation was observed, see Figure 5b. The comparison of results are also summarized in Table S3. Further comparison of PL intensity between Pb(Ac)_2_-doping and PbI_2_-doping CH_3_NH_3_PbI_3_ films can be observed in Figure 5c; the intensity was at the same level, indicating similar PL efficiencies for these two CH_3_NH_3_PbI_3_ materials. The PL traces of other Pb-rich samples with Pb(Ac)_2_ adjustment were also measured, and showed obvious enhancement in photostability (Supplementary Information Figure S4). The introduction of Pb(Ac)_2_ can effectively optimize the photostability of perovskite films fabricated via Pb-rich precursor solutions. Generally speaking, these improvements of PL properties, including PL efficiency and photostability, can be correlated with the defect properties in perovskite materials. This is because during the fabrication process it is relatively difficult to well control the defects in these materials. As reported, the formation energy of the defects can be significantly affected by the concentration of the defects. Thus, the relative amount of the precursors becomes important, which directly affects the concentration of the defects, resulting in the different properties of the perovskite materials. In addition, the surface defects of the materials can be passivated by many different agents, such as Lewis base and small molecules. The excess PbI_2_ and Pb(Ac)_2_ can also work for the passivation of the surface defects, resulting in the improvement of the PL efficiency and photostability. As a result, according to the results provided in our work, both CH_3_NH_3_I-rich and PbI_2_-rich CH_3_NH_3_PbI_3_ films with high photostability can be achieved by stoichiometric adjustment of precursors. That is, when preparing uniform photostable CH_3_NH_3_I-rich films with high PL efficiency, a small excess of CH_3_NH_3_I (less than 15%) and solvent engineering with Lewis base adduct method were required. In addition, as Pb-rich samples are also sometimes required to construct brilliant perovskite solar cells and LEDs structures, lead acetate can be introduced as the excess lead source, which can not only help to sustain the dense films and PL efficiency, but also can help to improve the photostability of the CH_3_NH_3_PbI_3_ films. Dense films can effectively isolate the short circuit effect, and high photostability can improve the durability during the work conditions.

## 4. Conclusions

In summary, we carefully investigated the effect of stoichiometric chemistry and solvent engineering on the fabricated CH_3_NH_3_PbI_3_ films for their morphology, coverage ratio, PL efficiencies, PL spectra, and photostability. When the films were fabricated under I-rich conditions, introduction of nonpolar solvent engineering during the spin casting and the addition of DMSO as a Lewis base helped to form dense CH_3_NH_3_PbI_3_ films with improved photostability and PL efficiencies. Under Pb-rich conditions, the films prepared by the same procedure showed a clearly decreased photostability, which can be avoided by substituting Pb(Ac)_2_ for PbI_2_. These results emphasize the significant influences of stoichiometric chemistry and solvent engineering on CH_3_NH_3_PbI_3_ films, and indicate a general strategy for the optimization of perovskite films.

## Data Availability

All data needed to evaluate the conclusions in the paper are present in the paper and/or the Supplemental Information. Additional data related to this paper may be requested from Y.T. (tyx@nju.edu.cn).

## Supplementary Materials

The following are available online at https://www.mdpi.com/2079-4991/11/2/405/s1, Figure S1: Stoichiometric effects on the photostability of CH_3_NH_3_PbI_3_ films fabricated via direct spin casting, Figure S2: Stoichiometric effects on the photostability of CH_3_NH_3_PbI_3_ films fabricated via solvent engineering methods, Figure S3: Stoichiometric effects on the photostability of the CH_3_NH_3_PbI_3_ films fabricated via solvent engineering with Lewis base adduct method, Figure S4: Stoichiometric effects on the photostability of Pb(Ac)_2_ doped CH_3_NH_3_PbI_3_ films fabricated via solvent engineering with Lewis base adduct method, Table S1: The comparison of the coverage via different fabrication methods with different stoichiometric precursors, Table S2: The comparison of the films’ photostability via different fabrication methods with different stoichiometric precursors, Table S3: The comparison of the films’ photostability fabricated via stoichiometric adjustment with different lead sources. And both films were prepared by solvent engineering with Lewis base adduct methods.
