# Supplementary material for: Photostable and Uniform CH3NH3PbI3 Perovskite Film Prepared via Stoichiometric Modification and Solvent Engineering
Source: Nanomaterials (Basel). 2021 Feb 5;11(2):405. doi: 10.3390/nano11020405 (PMC7915270; doi:10.3390/nano11020405)
Supplement: Supplementary file 1 [file nanomaterials-11-00405-s001.pdf]

nanomaterials

MDPI

Supplementary Materials

Photostable and Uniform $CH_3NH_3PbI_3$ Perovskite Film Prepared via Stoichiometric Modification and Solvent Engineering

Daocheng Hong [1], Mingyi Xie [2] and Yuxi Tian [2,*]

[1] Key Laboratory for Advanced Technology in Environmental Protection of Jiangsu Province, Yancheng Institute of Technology, Yancheng, Jiangsu, 224051, China; hdaocheng@gmail.com
[2] Key Laboratory of Mesoscopic Chemistry of MOE, School of Chemistry and Chemical Engineering, Nanjing University, Nanjing, Jiangsu, 210023, China; xmy@nju.edu.cn
* Correspondence: tyx@nju.edu.cn

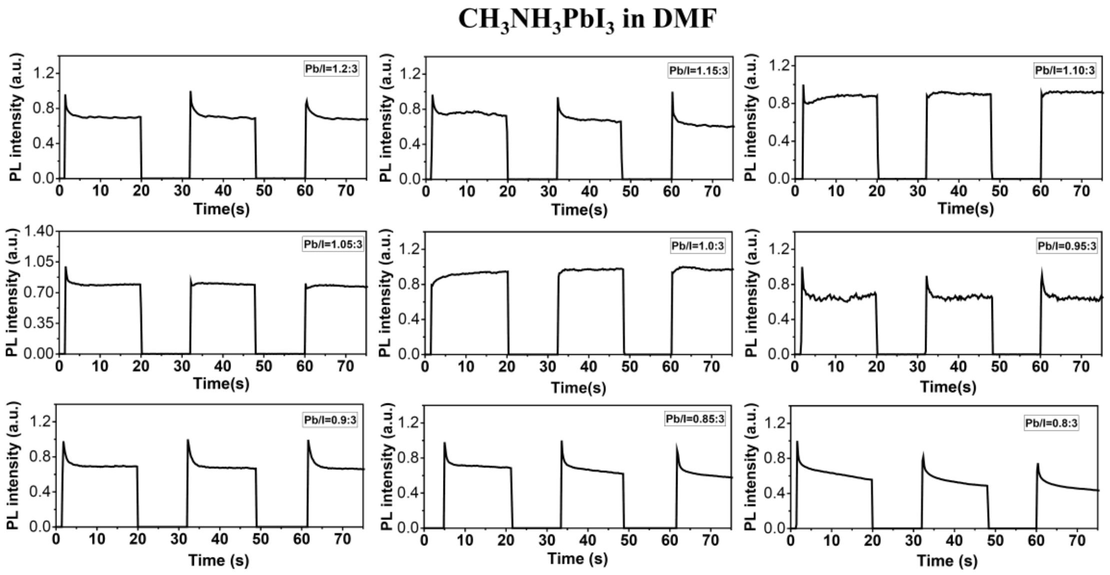

Figure S1. Stoichiometric effects on the photostability of $CH_3NH_3PbI_3$ films fabricated via direct spin casting.

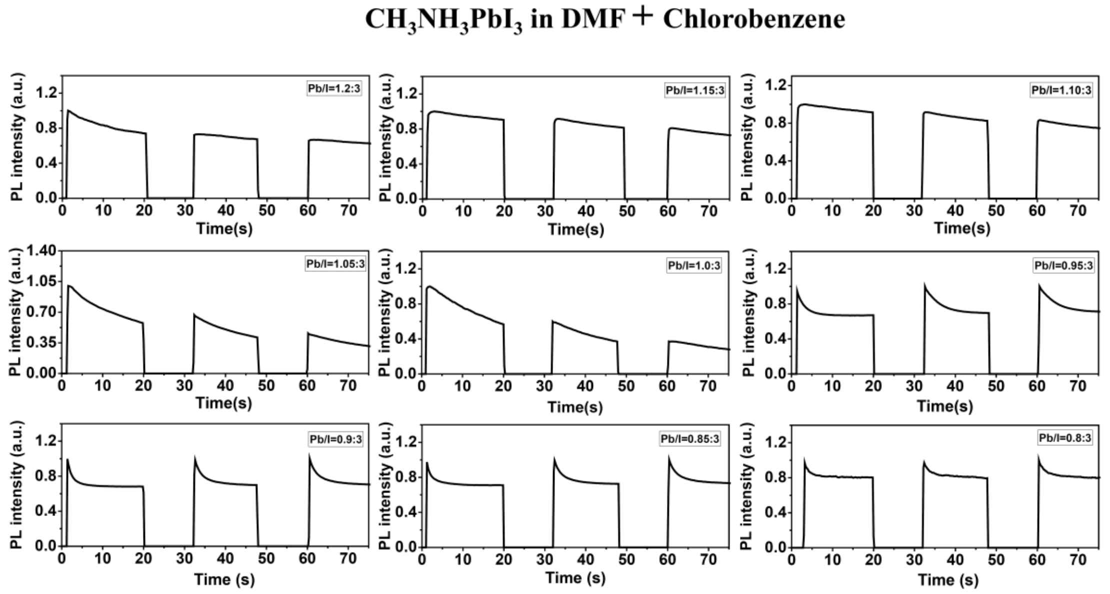

Figure S2. Stoichiometric effects on the photostability of $CH_3NH_3PbI_3$ films fabricated via solvent engineering methods.

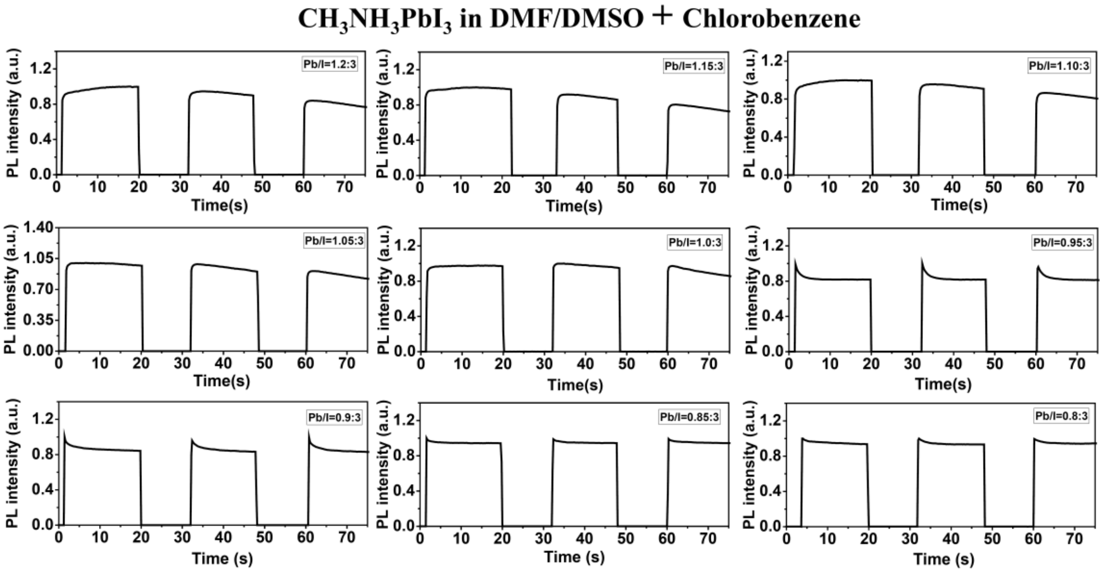

Figure S3. Stoichiometric effects on the photostability of the $CH_3NH_3PbI_3$ films fabricated via solvent engineering with Lewis base adduct method.

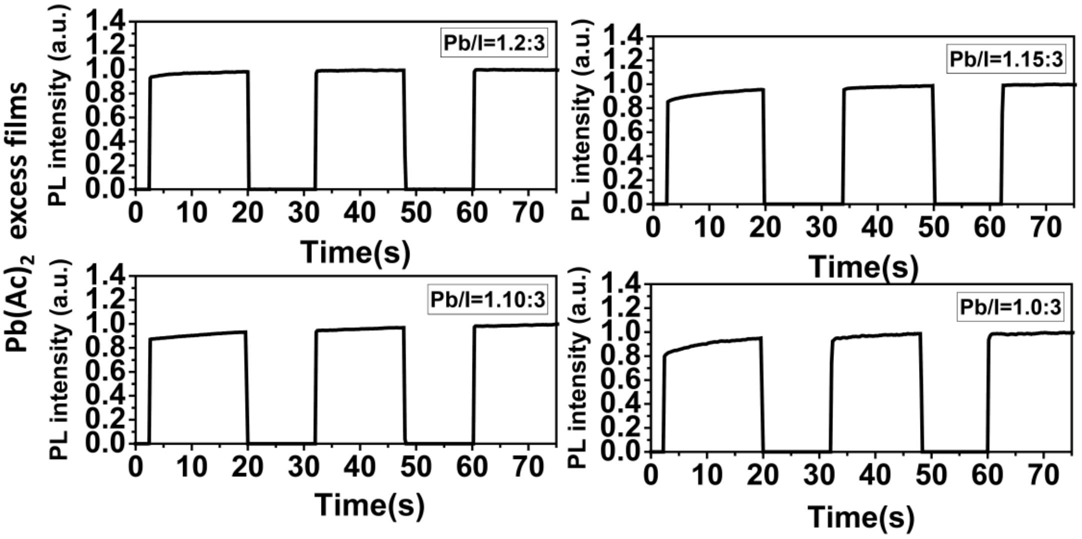

Figure S4. Stoichiometric effects on the photostability of $Pb(Ac)_2$ doped $CH_3NH_3PbI_3$ films fabricated via solvent engineering with Lewis base adduct method.

Table S1. The comparison of the coverage via different fabrication methods with different stoichiometric precursors.

Seq.	Pb:I	Solvent		
		DMF	DMF	DMF/DMSO
		Anti-Solvent		
		None	Chlorobenzene	Chlorobenzene
1	1.2:3	Poor	High	High
2	1.15:3	Poor	High	High
3	1.10:3	Poor	High	High
4	1.05:3	Poor	Medium	High

5	1.0:3	Poor	Medium	High
6	0.95:3	Poor	Medium	High
7	0.9:3	Poor	Poor	High
8	0.85:3	Poor	Poor	Medium
9	0.8:3	Poor	Poor	Medium

Table S2. The comparison of the films' photostability via different fabrication methods with different stoichiometric precursors.

Seq.	Pb:I	Solvent		
		DMF	DMF	DMF/DMSO
		Anti-Solvent		
		None	Chlorobenzene	Chlorobenzene
1	1.2:3	Stable	Fast degradation	Slow degradation
2	1.15:3	Stable	Fast degradation	Slow degradation
3	1.10:3	Stable	Fast degradation	Slow degradation
4	1.05:3	Stable	Fast degradation	Slow degradation
5	1.0:3	Stable	Fast degradation	Slow degradation
6	0.95:3	Stable	Stable	Stable
7	0.9:3	Stable	Stable	Stable
8	0.85:3	Stable	Stable	Stable
9	0.8:3	Stable	Stable	Stable

Table S3. The comparison of the films' photostability fabricated via stoichiometric adjustment with different lead sources. And both films were prepared by solvent engineering with Lewis base adduct methods.

Seq.	Pb:I	Lead Source	
		PbI_2	$Pb(Ac)_2$
1	1.2:3	Slow degradation	Stable
2	1.15:3	Slow degradation	Stable
3	1.10:3	Slow degradation	Stable
4	1.05:3	Slow degradation	Stable
